# The effect of bright light therapy on sleep in pregnant women with major depressive disorder– a randomized controlled trial

**DOI:** 10.1007/s00737-025-01573-2

**Published:** 2025-03-04

**Authors:** Milan Zarchev, Babette Bais, Julia S. Meijer, Hilmar H. Bijma, Bianca van der Zande, Annemarie I. Luik, Mijke P. Lambregtse-van den Berg, Astrid M. Kamperman

**Affiliations:** 1 Department of Psychiatry, Erasmus MC, Rotterdam, The Netherlands; 2 Epidemiological and Social Psychiatric Research Institute (ESPRi), Department of Psychiatry, Erasmus MC, Rotterdam, The Netherlands; 3 Department of Neonatology, Máxima Medical Center, Veldhoven, The Netherlands; 4 Department of Obstetrics and Gynaecology, Division of Obstetrics and Fetal Medicine, Erasmus MC, Rotterdam, The Netherlands; 5 Signify, Eindhoven, North Brabant The Netherlands; 6 Department of Epidemiology, Erasmus MC, Rotterdam, The Netherlands

**Keywords:** Sleep, Actigraphy, Light therapy, Pregnancy, Depression

## Abstract

**Purpose:**

Bright light therapy (BLT) is a potential treatment for depression during pregnancy, which may also improve sleep. We investigated whether BLT has an effect on self-reported and actigraphy-estimated sleep in pregnant women diagnosed with depressive disorder.

**Method:**

Sixty-seven pregnant women with a DSM-5 diagnosis of depressive disorder during pregnancy were randomly allocated to treatment with BLT (9,000 lx, 5,000 K) or dim red light therapy (DRLT, 100 lx, 2,700 K), which is considered placebo. For six weeks, both groups were treated daily at home for 30 min upon awakening. Follow-up took place at various time points. We collected data on sleep with the Pittsburgh Sleep Quality Index and with actigraphy wearables.

**Results:**

We found no statistically significant differences in treatment groups across any of the sleep parameters measured, namely sleep efficiency, duration, onset latency, fragmentation, and total sleep health as measured by self-report and actigraphy. Moreover, we observed no overall improvements in sleep during the treatment period.

**Conclusions:**

The results suggest that any potential therapeutic effects of BLT might have on sleep are too small for the current study to detect.

**Clinical trial number:**

NTR5476; November 5th, 2015

**Supplementary Information:**

The online version contains supplementary material available at 10.1007/s00737-025-01573-2.

## Highlights

Bright light therapy had no detectable effect on subjective sleep health in pregnant women with depression.

Bright light therapy also had no detectable effect on actigraphy-estimated sleep parameters.

Other forms of intervention may be more suited to address highly prevalent sleep problems in this population.

**Supplementary Information:**

The online version contains supplementary material available at 10.1007/s00737-025-01573-2.

## Introduction

Approximately half of all pregnant women suffer from significant sleep problems, especially in the third trimester (Yang et al. 2020). These problems do not only affect the health of the mother, but also that of their (unborn) child (Lyu et al. 2020; Hoyniak et al. 2024; Jiang et al. 2024; Zhong et al. 2018; Wang and Jin 2020; Warland et al. 2018). One of the strongest associations with maternal sleep problems is worsening of perinatal maternal health (Okun 2015; Bai et al. 2024). Among pregnant women with depression, which affects approximately 11–15% of pregnant women (Woody et al. 2017; Liu et al. 2022), sleep problems are even more prevalent (Reichner 2015). The most prevalent sleep problems in pregnancy include breathing-related sleep problems, restless legs syndrome, and insomnia (Nodine and Matthews 2013). Amongst depressed pregnant women specifically, probelms also include difficulty falling asleep, sleep continuation, early awakening, fragmentation and disruption of daily activities (Okun et al. 2011). Since both perinatal depression and sleep problems negatively impact the mother and the (unborn) infant, it is important to study non-pharmacological treatment options that are efficient and safe. A possible alternative is bright light therapy (BLT), which has been found to be effective in the treatment of seasonal affective disorder and non-seasonal depression (Al-Karawi and Jubair 2016; Martensson et al. 2015; Perera et al. 2016). Various studies have investigated the effectiveness of BLT for treating peripartum depression as well (Corral et al. 2007; Epperson et al. 2004; Oren et al. 2002; Wirz-Justice et al. 2011). While these studies found indications of a positive effect of BLT, their sample sizes were small, thus limiting their power. A recent study executed by our group studied a larger sample size, had a longer follow-up, and studied infant outcomes as well (Bais et al. 2020). We found that mean depression scores decreased for both the intervention with BLT and the intervention we considered as placebo, without a statistically significant difference on depressive symptoms between the two treatment arms (Bais et al. 2020). Earlier, it has been shown that BLT can improve sleep quality as well and is able to decrease sleep onset latency and induce a phase advance in get-up time and bedtime (Maanen et al. 2016). Considering the relationship between sleep and depression, also in the peripartum period (Okun et al. 2011; Coo Calcagni et al. 2012; Kamysheva et al. 2010; Lewis et al. 2018; Park et al. 2013), light therapy may not only improve sleep quality but also reduce depressive symptoms through better sleep.

In the present study, we investigate whether BLT has an effect on self-reported and actigraphy-measured sleep in pregnant women diagnosed with depression.

## Materials and methods

The present study made use of previously collected data from the Bright Up study, which assessed the effects of BLT on depressive disorder during pregnancy.

### Design

The Bright Up study was a randomized, double-blind, placebo-controlled clinical trial studying the effects of light therapy on depression during pregnancy (Bright Up, NTR5476, http://www.trialregister.nl, registered November 5th, 2015) (Bais et al. 2020). The complete study protocol can be found elsewhere (Bais et al. 2016). In this study, we aimed to study the effectiveness of BLT in pregnant women diagnosed with major depressive disorder, compared to a control condition (DRLT– dim red light therapy).

### Ethics

All procedures performed involving human participants were in accordance with the ethical standards of the institutional and/or national research committee and with the 1964 Helsinki declaration and its later amendments or comparable ethical standards. Written informed consent was obtained from all participants. The study protocol and later amendments were approved by the medical ethical committee of the Erasmus University Medical Centre, Rotterdam, The Netherlands (registration number MEC-2015-731).

### Participants

The Bright Up study included women (18–45 years) who were 12–32 weeks pregnant and had a DSM-5 diagnosis of depressive disorder, confirmed by a Structured Clinical Interview for DSM disorders (SCID) by one trained assessor (First et al. 2002). The exclusion criteria were as follows: insufficient proficiency in Dutch or English, multiple pregnancies, current use of antidepressants shorter than 2 months, a lifetime diagnosis of bipolar I or II disorder, any psychotic episode, current substance abuse, current primary anxiety disorder, recent history of a suicide attempt, current shift-work, somatic and/or obstetric conditions that override study participation, previous treatment with BLT, and eye conditions, eye diseases, or recent eye surgery.

Participants were recruited via general practitioner and midwife practices, hospitals, and via the outpatient psychiatric clinic and the obstetric outpatient clinic at the Erasmus University Medical Center. Women could also enroll themselves via advertisements on (social) media platforms. This resulted in the enrolment of 283 women.

Finally, 67 women were included in the original study. A flow-chart of the current study can be found in Supplementary Fig. 1. Of these women, 33 were allocated to the BLT-group and 34 were allocated to the DRLT-group. Of 3 women, no PSQI were assessed at inclusion and of 34 women, actigraphy data was not complete. This was due to the women giving birth close to starting the actigraphy measurement (*n* = 12), refusing without giving reason (*n* = 5), skin irritation from the actiwatch band (*n* = 4), logistic barriers to recover the actiwatch (*n* = 3), device failure (*n* = 2) or a combination of various reasons (*n* = 6).

### Method

Before inclusion, participants were instructed about the study and its aims, and gave their written informed consent. A baseline interview to investigate demographic information, obstetric information, psychiatric information, medication use, and somatic conditions was administered by telephone. In this interview, participants were also screened with the SCID to assess the diagnosis of depressive disorder and potential comorbidities, such as anxiety disorder and posttraumatic stress disorder. Relevant other data were collected during this interview, including the Structured Interview Guide for the Hamilton Depression Rating Scale– Seasonal Affective Disorders version (SIGH-SAD) to assess depressive symptoms (Williams et al. 1988). This is a 29-item structured interview that is used to investigate depressive symptomatology and is commonly used in light therapy trials. It consists of 21 items of the Hamilton Rating Scale for Depression (HRSD) and 8 atypical items. Blinded assessors administered the SIGH-SAD by telephone at various follow-up moments.

After baseline measurements (T0) and obtaining informed consent, participants were 1:1 randomized into one of two groups: BLT (9,000 lx, 5,000 K) or DRLT (100 lx, 2,700 K). The box was a research modified version of Phillips model HF3419. The installation of the box was conducted by the researcher team. A custom made scaffolding was constructed for each participant so that the height could be adjusted and glare avoided. Detailed instructions were provided. Compliance was self-reported and monitored on a weekly basis. Following randomization, participants were treated daily for six weeks with light for 30 min per day within 30 min of habitual wake-up time. During this intervention period, weekly questionnaires were used to assess sleep quality. Additionally, participants were instructed to wear an actiwatch for the full intervention period and two weeks after that, resulting in 8 weeks of actigraphy-estimated sleep measures.

### Materials

The Bright Up study collected various materials, such as questionnaires, body material (hair, saliva, urine), actigraphy, and medical files. The present study will only assess the actigraphy and the Pittsburgh Sleep Quality Index (PSQI) as outcomes.

#### Self-reported sleep

The PSQI was assessed to investigate self-reported sleep quality (Buysse et al. 1989). As such, it was one of the secondary outcome measures of the current study. The questionnaire assesses sleep quality and disturbances in the last month, and assesses seven subscales: subjective sleep quality, sleep onset latency, total sleep duration, sleep efficiency, sleep disturbances, use of sleep medication, and daytime dysfunction during the past month. The PSQI yields a total score of 0 to 21, a higher score indicating a worse quality of sleep. In the current study, we used an adapted version of the PSQI to assess self-reported sleep weekly during the intervention period. The PSQI could be filled in at home via a link sent by e-mail. In the present study, next to the total PSQI score, three subscales were studied (sleep efficiency, sleep onset latency, and sleep duration) by analogy of the actigraphically measured parameters. Other than the PSQI, no further sleep diaries were filled out.

#### Actigraphy-estimated sleep

Women were instructed to wear the actiwatch on their non-dominant wrist for 8 weeks at baseline and during the intervention period. Actiwatch data was analyzed using Respironics Actiware 5.0 (ActiWatch Spectrum, Philips Respironics, Pittsburgh, USA). The following actigraphic sleep measures were used: sleep duration, sleep onset latency, sleep efficiency, and sleep fragmentation. Sleep duration is the total sleep duration measured in hours and minutes. Sleep onset latency is the time in bed until sleep, measured in hours and minutes. Sleep efficiency is the percentage of time spent asleep while in bed. Sleep fragmentation is the percentage of mobility phases during sleep. Lower total sleep time, higher sleep latency, lower sleep efficiency, and higher sleep fragmentation indicate more sleep problems.

Mean weekly actiwatch scores were calculated based on three or more weekdays per observational period in line with best methodological recommendations from the sleep literature (Aili et al. 2017; Ancoli-Israel et al. 2003; Berger et al. 2008; Morgenthaler et al. 2007; Ravesteyn et al. 2014). We removed data from weekdays that coincided with daylight saving or public holidays. By way of sensitivity analyses, we also calculated mean weekly actiwatch scores based on both weekdays and weekend days over a minimum of five days or more. These results are presented in Supplementary Table 1.

#### Combination of self-reported and actigraphy-estimated sleep

A weekly measure of sleep health was finally calculated using both the PSQI and the actigraphy parameters following the SATED model (Buysse 2014). Briefly, the SATED score quantifies five dimensions of sleep health (Satisfaction, Alertness, Timing, Efficiency, Duration) as “poor” = 0 or “good” = 1 based on adult sleep norm scores. We followed previous literature in determining the cut-offs for each dimension (Lichstein et al. 2003; Hirshkowitz et al. 2015; Walch et al. 2016; Wallace et al. 2019). Sleep satisfaction was considered good if the PSQI item “How would you rate your sleep quality overall?” was responded to as “Fairly good” or “Very good”. Alertness was considered good if the PSQI item “How often do you have trouble staying awake while driving, eating, or doing social activities?” was responded to as “Less than once in the past week”, or “Not during the past week”. Timing was classified as good if the average weekly midpoint of sleep derived from actigraphy was between 2AM and 4AM. Efficiency was classified as good if average actigraphy-estimated sleep efficiency was more than 85%. Finally, duration was classified as good if average sleep duration was actigraphically estimated to be between 6 and 8 h of sleep. The final SATED score sums all indices together into a single score ranging from 0 to 5, with higher scores indicating better sleep health.

### Statistical analysis

The outcome measures of the present study were self-reported and actigraphy-estimated sleep parameters over the course of the intervention period. These included sleep efficiency, sleep duration, and sleep onset latency, which were estimated using both self-report and actigraphy methods. We included the PSQI total score as a self-report only outcome. Furthermore, the sleep fragmentation index was included as an actigraphy-measured only outcome. Finally, self-report and actigraphy data was combined to calculate the SATED total score.

The effects of BLT compared to DRLT on sleep were investigated using generalized linear mixed model analyses, using a random intercept and random time slope for each participant. As fixed effects, we included allocation to experimental condition, assessment (time), and the interaction term between allocation and time, as well as mean-centered baseline score to adjust for individual differences with regard to the outcome at baseline. We also adjusted for age, education, parity, chronotype (as assessed by the Munich Chronotype Questionnaire (Roenneberg et al. 2003), and seasonal depressive symptoms (as measured by the Structured Interview Guide for the Hamilton Depression Scale—Seasonal Affective Disorder) to further increase the precision of estimates (Lingsma et al. 2010).

We report on overall effects across all time points as implemented in the R package “marginaleffects” (Arel-Bundock et al. 2024). We also report on significance of allocation * time interaction parameter to test for time-varying effects of allocation. Finally, predicted means and corresponding 95% confidence intervals at each time points are plotted for visual representation of the time-varying effects of allocation.

No data were available on seasonal depressive symptoms for 3 participants. Mean replacement was used for those cases. Analyses were conducted using and R (version 4.1.3 (Team. 2021),. Code used for the analyses in this study can be found online at https://osf.io/hr49p.

## Results

### Demographic and clinical characteristics

Baseline participant characteristics of the two treatment arms are shown in Table 1. Briefly, average age of the mothers was 32.0 years, they were mostly of Dutch national origin (80.6%) and most were married (96.9%).

**Table 1:** Overview of participant characteristics included in analyses at inclusion

	BLT (*n* = 31)	DRLT (*n* = 33)
**Age in years, mean (SD)**	32.0 (4.4)	31.9 (5.4)
**Gestational age in weeks, mean (SD)**	20.6 (6.2)	19.9 (6.3)
**Country of birth**		
Dutch	25 (80.6%)	25 (75.8%)
Other	6 (19.4%)	8 (24.2%)
**Marital status**		
Married or cohabiting	31 (100%)	31 (94.0%)
Committed relationship, not cohabiting	0 (0%)	1 (3.0%)
Single	0 (0%)	1 (3.0%)
Education*		
Elementary or (pre-)vocational education	9 (29.0%)	12 (36.3%)
Higher professional education	8 (25.8%)	11 (33.3%)
(Pre-)academic education	14 (45.1%)	10 (30.3%)
**Number of older children**		
None	14 (45.2%)	20 (60.6%)
One older child	13 (41.9%)	9 (27.3%)
More than one child	4 (12.9%)	4 (12.1%)
BMI in kg/m^2^, mean (SD)	25.6 (4.6)	26.5 (5.3)
**Planned pregnancy**	21 (67.7%)	21 (63.6%)
**Antidepressant medication use**	3 (9.7%)	5 (15.2%)
**Sleep medication use**	2 (6.5%)	2 (6.1%)
**Psychotherapy**	13 (41.9%)	16 (48.5%)
**Comorbidities**		
None	17 (54.8%)	13 (39.4%)
One comorbid disorder	9 (29.0%)	13 (39.4%)
More than one disorder	5 (16.2%)	7 (21.2%)
**Chronotype**		
Early (extreme, moderate, and slight)	19 (61.3%)	25 (75.7%)
Normal	1 (3.2%)	1 (3.0%)
Late (extreme, moderate, and slight)	4 (12.9%)	1 (3.0%)
**Duration of depression in weeks, mean (SD)**	25.7 (16.9)	46.3 (123.6)
**Depressive episodes in past**		
None	12 (38.7%)	11 (33.3%)
One prior episode	8 (25.8%)	13 (39.4%)
More than one episode	11 (35.5%)	9 (28.3%)
**Depressive symptoms at baseline, mean (SD)**	16.9 (5.2)	16.8 (5.6)

BLT = bright light therapy;

DRLT = dim red light therapy;

SD = standard deviation

* Categories roughly correspond to: Primary & Secondary Education, Applied Higher Education, and Academic Higher Education in internationally recognized terms.

### Actigraphy-estimated and self-reported sleep at baseline

The results from the actigraphy-estimated and self-reported sleep parameters differed in that self-reported sleep efficiency and duration was lower compared to the actigraphy-estimated parameters (Table 2). Likewise, sleep latency was reported to be higher when measured via self-report compared to actigraphy obtained methods. The mean PSQI score of the entire study group is 9.7, indicating that women on average rated their sleep quality as poor.

**Table 2:** Self-reported and actigraphy-estimated sleep parameters the intervention (BLT) and control (DRLT) condition at inclusion

**Self-reported sleep parameters**	Total (*n* = 64)	BLT (*n* = 31)	DRLT (*n* = 33)
Efficiency in %, (SD)	73.4 (16.0)	73.3 (14.9)	73.5 (17.1)
Duration in hh: mm, mean (SD)	06:50 (01:37)	06:46 (01:24)	06:55 (01:49)
Onset latency in hh: mm, mean (SD)	00:40 (00:35)	00:40 (00:38)	00:40 (00:33)
Total PSQI score (range 0–21), mean (SD)	9.7 (3.5)	9.4 (3.2)	10 (3.7)
**Actigraphy-estimated sleep parameters**	Total (*n* = 35)	BLT (*n* = 17)	DRLT (*n* = 18)
Efficiency in %, mean (SD)	85.1 (4.0)	85.6 (3.8)	84.5 (4.2)
Duration in hh: mm, mean (SD)	07:39 (00:34)	07:34 (00:46)	07:34 (01:01)
Onset latency in hh: mm, mean (SD)	00:15 (00:10)	00:17 (00:10)	00:15 (00:11)
Fragmentation index in %, mean (SD)	30.4 (8.6)	29.9 (10.5)	30.9 (6.6)
**Combined actigraphy and self-report parameters**	Total (*n* = 35)	BLT (*n* = 17)	DRLT (*n* = 18)
Total SATED score (range 0–5), mean (SD)	2.89 (1.00)	2.93 (1.06)	2.84 (1.06)

BLT = bright light therapy;

DRLT = dim red light therapy;

PSQI = Pittsburgh Sleep Quality Index

SATED = Satisfaction, Alertness, Timing, Efficiency, Duration

SD = standard deviation

### Effects of bright light therapy on sleep and activity

**Fig. 1:**
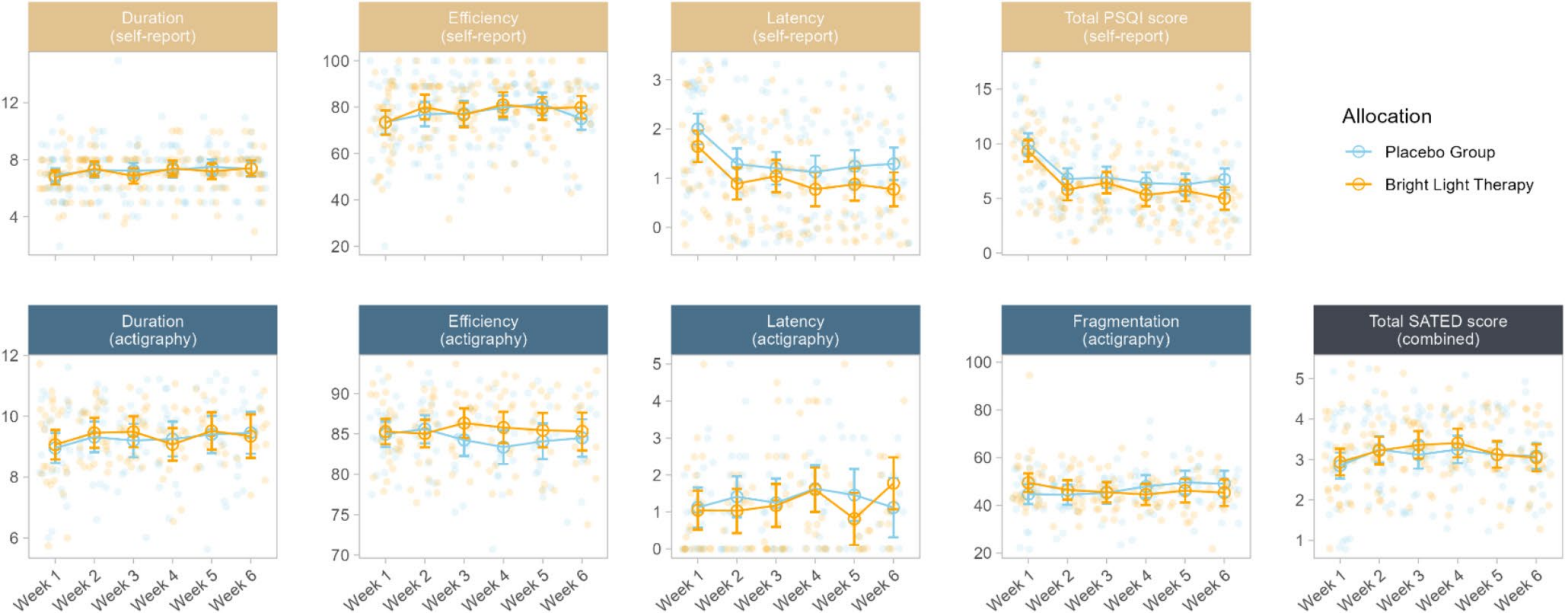
Averaged weekly sleep outcomes during the treatment period, as measured by the self-reported pittsburgh sleep quality index and actigraphy-obtained parameters. Orange shows the group treated with bright light therapy and blue shows the placebo group

Table 3 presents the overall effects of allocation on sleep parameters and its interaction with time over the course of treatment. There were no overall effects of light therapy for any of the included sleep parameters (all *p* >.078). When investigating the interaction between allocation and time, only actigraphy-measured sleep efficiency showed a significant trend over time (*p* =.034). Figure 1 visualizes predicted values for the placebo and BLT groups. A higher average sleep efficiency was observed in week 3 and 4 for the BLT group, before the difference disappeared again in week 5 and 6. This interaction with time did not remain significant after FDR adjustment for multiple comparisons (adjusted *p* =.226).

**Table 3:** Overall effects of allocation on self-reported and actigraphy-estimated quality of sleep through the treatment period and allocation X time interaction

Outcome	Unstandardized beta [95%CI]^1^	*p*-value	Time interaction *p*-value
**Self-reported sleep parameters**			
Duration	0.06 [-0.49, 0.61]	0.836	0.097
Efficiency	2.16 [-3.44, 7.77]	0.449	0.258
Latency	-0.23 [-0.59, 0.13]	0.211	0.197
Total PSQI score	-0.94 [-1.98, 0.10]	0.078	0.200
**Actigraphy-estimated sleep parameters**			
Duration	0.47 [-0.16, 1.10]	0.147	0.066
Efficiency	0.41 [-2.00, 2.81]	0.741	0.034
Latency	-0.04 [-0.45, 0.36]	0.833	0.109
Fragmentation	1.38 [-4.19, 6.95]	0.628	0.126
**Combined self-reported and actigraphy-estimated sleep parameters**			
Total SATED score	0.03 [-0.29, 0.35]	0.835	0.682

^*1*^ Adjusted for age, education, parity, chronotype and seasonal depressive symptoms

BLT = bright light therapy; DRLT = dim red light therapy;

PSQI = Pittsburgh Sleep Quality Index

SATED = Satisfaction, Alertness, Timing, Efficiency, Duration

## Discussion

We studied the secondary outcome of an RCT, where we evaluated the effects of BLT on sleep and activity in pregnant women suffering from depressive disorder, compared to the placebo condition DRLT. We studied longitudinal self-reported and actigraphy-estimated sleep parameters in these women during pregnancy.

In the present study, we did not find any statistically significant differences in the included sleep parameters, suggesting that BLT does not improve sleep in pregnant women diagnosed with depressive disorder at a magnitude we could detect. In an earlier study based on the same data, we reported no improvement in depressive symptoms either in the current sample of pregnant women (Bais et al. 2020). Three other trials investigated the effect of bright light therapy on sleep during pregnancy, which were meta-analyzed to indicate an overall small but positive effect on sleep (Li et al. 2023). Of note, one of those studies investigated women with perinatal depression as well and reported an improvement in sleep following BLT treatment (Donmez et al. 2022). While Donmez et al. (2022) used the PSQI as an outcome measure of subjective sleep like in the current study, there were other key differences. Follow-up time was shorter (three weeks vs. the current six), BLT was administered for longer (45 min vs. current 30) and the sample size was smaller (12 per group). Sleep quality was found to improve both for the active treatment and placebo group, but significantly more so under BLT. In contrast, we found that both treatment groups were relatively stable on both objective and subjective sleep metrics. This suggests that the difference in results is not due to the treatment procedure, but possibly due to Donmez et al. (2022) continuing follow-up after delivery which could produce substantial changes in sleep.

The null results here may be explained by the fact that sleep problems are highly prevalent during pregnancy and increase during the course of pregnancy (Yang et al. 2020). This was reflected further by the very early chronotype among almost all participants, despite almost complete lack of sleep medication use in the sample. Furthermore, persistent sleep problems commonly co-occur with depressive symptoms (Okun et al. 2011). The interaction of biological and environmental changes during pregnancy with depressive symptoms might exert too strong of an effect on sleep for BLT to make a large difference. Possibly, sleep may be influenced by other factors that may differ between women, such as trouble sleeping due to lower back pain, restless leg syndrome, repositioning, urinary frequency or other physio-mechanical complaints related to pregnancy (Silvestri and Arico 2019). Any of these factors might be more appropriate targets for future interventions aimed at improving sleep in depressed pregnant women.

There are challenges in comparing the current results to the wider prior empirical literature. Light therapy trials in pregnant women with depressive disorder so far have not comprehensively studied a wide range of sleep parameters (Epperson et al. 2004; Oren et al. 2002; Wirz-Justice et al. 2011). However, outside of pregnancy some effects of light therapy on these parameters have been found in patients with depression. For example, a study among patients suffering from seasonal affective disorder showed that BLT decreased sleepiness (Ivanova et al. 2016). A light therapy trial among patients with Parkinson’s disease suffering from depression showed that self-reported sleep quality improved in both the BLT and the placebo group, with a larger improvement in patients treated with BLT (Rutten et al. 2019). Among elderly patients suffering from depression, BLT seemed to increase sleep efficiency, advance get-up time, and decrease total sleep time (Lieverse et al. 2011). In contrast, we found no improvement in any sleep parameters, indicating that the period of pregnancy might add additional barriers when comparing to clinical populations in which BLT was effective.

## Strengths and limitations

The Bright Up study has several notable strengths. The current investigation is to date the largest RCT to study the effects of BLT in pregnant women diagnosed with depressive disorder. Moreover, the follow-up period was among the longest in any randomized sleep study focused on pregnant women. Furthermore, we report on actigraphy-estimated sleep parameters in contrast to most previous literature focusing solely on self-report measurements of sleep. The self-report and actigraphy measurements we used are standard instruments in sleep research, which allows for straight-forward comparisons with future sleep studies in this population.

The main limitation of the Bright Up study is not reaching the original sample target of 150 participants. Consequently, we could only detect larger treatment effects than originally planned. Thus, the current study cannot reject a potentially small effect of BLT on sleep. Despite this the current study is nevertheless recruited as many participants per group as the largest BLT trial studying perinatal sleep to date (Verma et al. 2023). Incomplete actigraphy measurements caused an additional proportion of missing data in our study sample. The long intervention period and follow-up time could have contributed to the burden of providing complete data. While the treatment effects for most sleep outcomes clustered around a zero difference, increased statistical power could have allowed us to detect small differences between the groups. However, the clinical relevance of such small differences is questionable. However, variation in habitual wake-up time could also contribute to a weakened effect of BLT. It further remains to be studied the placebo effects of any light therapy compared to no treatment at all.

## Conclusions

In the present study, we did not find a statistically significant effect of bright light therapy on a wide range of sleep parameters. These findings were consistent, regardless of whether sleep was operationalized via self-report questionnaires or actigraphy-estimated measurements. Moreover, we did not see unequivocally improvements in sleep during the treatment period.

## Electronic Supplementary Material

Below is the link to the electronic supplementary material.

Supplementary Materials
