# Supplementary material for: The effect of bright light therapy on sleep in pregnant women with major depressive disorder– a randomized controlled trial
Source: Arch Womens Ment Health. 2025 Mar 4;28(5):1261–9. doi: 10.1007/s00737-025-01573-2 (PMC12436560; doi:10.1007/s00737-025-01573-2)
Supplement: Supplementary file 1 — Supplementary Materials [file 737_2025_1573_MOESM1_ESM.docx]

**Supplementary Figure 1.** Flow-chart of the Bright Up study.


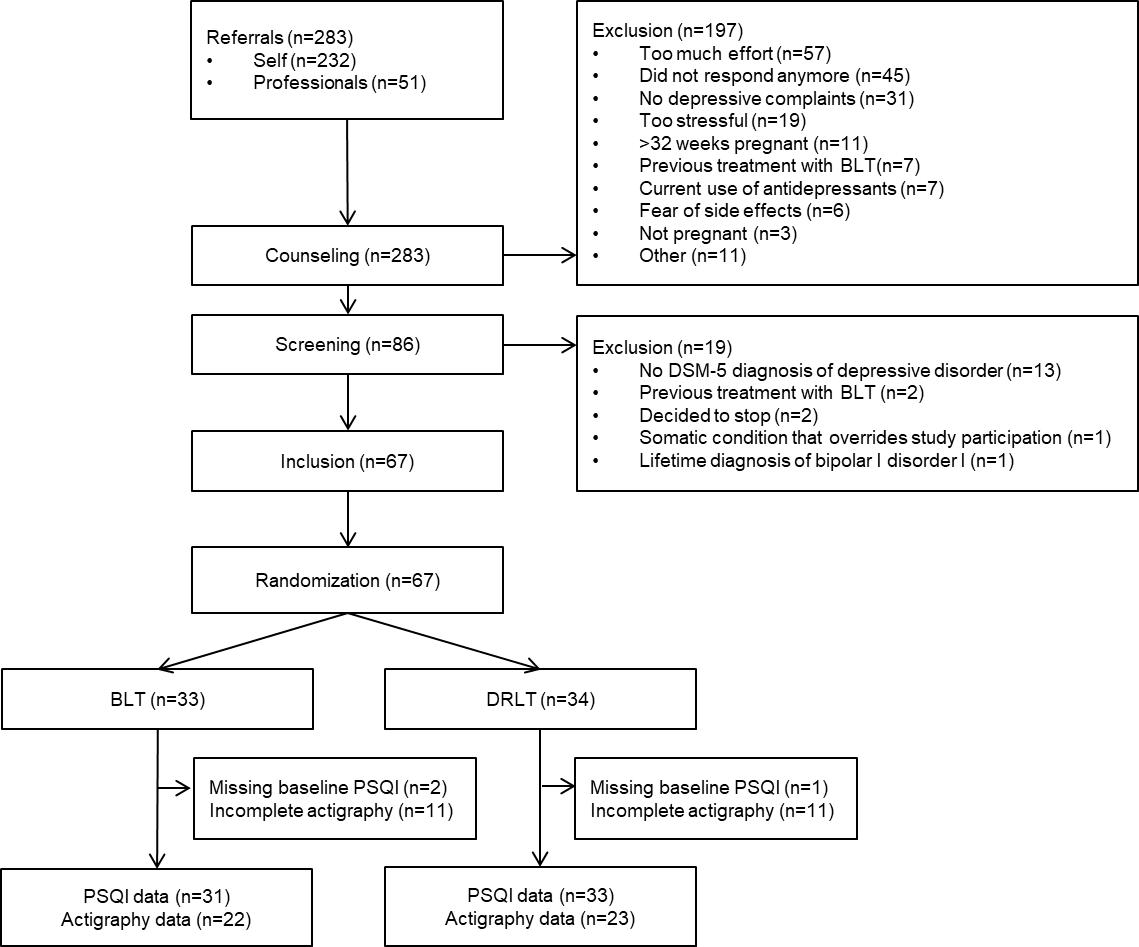


| **Supplementary Table 1**. Effects of allocation on objective parameters of sleep, as measured by actigraphy, through the treatment period: allocation x time interaction term, based on full weeks (Monday through Sunday). Analyses are both crude and adjusted. | | |
| --- | --- | --- |
| **Outcome** | **β (95% CI)** | **p-value** |
| Sleep efficiency | 0.23 (-0.40 – 0.85) | 0.474 |
| Sleep duration | 4.17 (-5.05 – 13.38) | 0.373 |
| Sleep onset latency | 0.35 (-1.66 - 2.37) | 0.730 |
| * Analyses adjusted for SIGH-SAD score at baseline, gestational age at baseline, level of education as a proxy for socioeconomic status, parity, medication use (benzodiazepines and antidepressants), and chronotype. | | |
